# CHOOSING TO FORGO: LIFE-SUSTAINING TREATMENT DECISIONS FOR SELVES AND RELATIVES AMONG TAIWANESE ADULTS

**DOI:** 10.1093/geroni/igae098.3058

**Published:** 2024-12-31

**Authors:** Duan-Rung Chen, Su-I Hou, Yuchi Young

**Affiliations:** National Taiwan University, Taipei, Taipei, Taiwan (Republic of China); The University of Central Florida, Orlando, Florida, United States; State University of New York at Albany, Albany, New York, United States

## Abstract

**Purpose:**

Since its implementation in 2019, less than 2% of adults in Taiwan have signed Advance Directives (ADs) under the Patient Right to Autonomy Act (PRAA). This research explores how the public views the PRAA and its effect on the decision to decline life-sustaining treatments (LSTs) in hypothetical end-of-life scenarios. Method: In August-November 2022, an online survey was administered to 3,922 adults in Taiwan, of which approximately 1,488 (37%) were familiar with the PRAA. These 1488 respondents completed questions regarding their perceptions of the PRAA. They responded to questions about three hypothetical end-of-life situations: terminal cancer, irreversible severe brain damage, and terminal dementia.

**Results:**

Over half of the participants declined to receive LSTs for themselves and chose similarly but less for their family members. A specific difference was noted in choices concerning terminal dementia, showing a greater reluctance to withhold LSTs, especially for parents, than for themselves. There were gender-based differences, with women more likely to choose against LSTs for themselves in various situations. In contrast, gender did not affect decisions made for family members. The perception of the PRAA and the degree of ACP engagement significantly influenced the refusal of LSTs for both the participants and family members. A positive view of the PRAA was significantly linked to decisions against LSTs for spouses.

**Conclusion:**

These findings underscore the complexity of decisions around LSTs, influenced by personal beliefs, gender, legal perceptions, and engagement for advance care planning.

